# 

**Published:** 1851-02

**Authors:** 


### WESTERN JOURNAL

OF

### MEDICINE AND SURGERY.

### EDITORS.

---

LUNSFORD P. YANDELL, M. D.
PROFESSOR OF PHYSIOLOGY AND PATHOLOGICAL ANATOMY IN THE UNIVERSITY OF LOUISVILLE.

AND

THEODORE S. BELL, M.D.

### *Receipts of Medical Journal for January,* 1851.

| | |
|---|---|
| Dr. A. Moore, Warrenton, Ala. | $20 00 |
| Dr. Godfrey, Kalida, O. | 10 00 |
| J. Barnes, McMinville, Tenn. | 10 00 |
| W. H. Baldwin, Finley, O. | 15 00 |
| I. Swain, Ballardsville, Ky. | 10 00 |
| S. Thompson, Albion, Ill. | 10 00 |
| A. C. McDonald, Big Creek, Tenn. | 20 00 |
| W. Schooley, Somerton, O. | 15 00 |
| J. B. Cross, Carpentersville, Ind. | 5 00 |
| M. Hovy, Lodi, O. | 5 00 |
| J. Slackhouse, Poplar Grove Ky. | 5 00 |
| G. O. Pond, Columbus, Ill. | 5 00 |
| —— Springs, Rough and Ready, Tenn. | 5 00 |
| D. H. Davis, Jackson, Mo. | 5 00 |
| J. L. Canterbury, Mexico, Mo. | 10 00 |
| G. Holtz, Canton, O. | 10 00 |
| J. B. Sabastian, Centrevill, Tenn. | 5 00 |
| —— McClurken, Halesville, S. C. | 5 00 |
| M. Brice, Youngsville, S. C. | 5 00 |
| J. C. Cook, Newport, Ind. | 10 00 |

The Western Journal of Medicine and Surgery is published monthly by the undersigned, at the corner of Main and Fifth streets, Louisville, at $5 per annum, payable in advance. Each number contains 96 pages, making two volumes in the year of more than 550 pages each.

Contributions to its pages by the Physicians of the Valley of the Mississippi, addressed to the Editors, are respectfully solicited.

Letters on business, to be addressed, postage paid, to the publishers.

PRENTICE & CO.

